# Green Tea: A Novel Perspective on the Traditional Plant’s Potential in Managing Periodontal Diseases

**DOI:** 10.3390/ph18030409

**Published:** 2025-03-14

**Authors:** Magdalena Paczkowska-Walendowska, Jan Grzegorzewski, Jakub Kwiatek, Marta Leśna, Judyta Cielecka-Piontek

**Affiliations:** 1 Department of Pharmacognosy and Biomaterials, Poznan University of Medical Sciences, Rokietnicka 3, 60-806 Poznan, Poland; j.grzegorzewski2001@gmail.com (J.G.); jpiontek@ump.edu.pl (J.C.-P.); 2 Science-Bridge Sp. z o.o., Chociszewskiego 24/8, 60-258 Poznan, Poland; 3 Kwiatek Dental Clinic Sp. z o.o., Kordeckiego 22, 60-144 Poznan, Poland; jakubkwiatek@klinikakwiatek.pl (J.K.); martalesna@klinikakwiatek.pl (M.L.)

**Keywords:** green tea, traditional plant, oral infections

## Abstract

Green tea (*Camellia sinensis*) exhibits significant potential in oral health due to its antioxidant, anti-inflammatory, and antimicrobial properties. This review explores its role in managing periodontal disease, a common condition characterized by inflammation, microbial imbalances, and tissue destruction. The primary bioactive components, particularly epigallocatechin-3-gallate (EGCG), contribute to green tea’s therapeutic effects by inhibiting bacterial adhesion, modulating inflammatory pathways, and reducing oxidative stress. Clinical studies suggest green tea improves periodontal health by reducing pocket depth, inflammation, and bleeding. It can serve as an adjunct to conventional therapies, including scaling and root planing, and be incorporated into oral care products such as mouthwashes and dentifrices. Furthermore, green tea presents a natural alternative to chemical agents like chlorhexidine, potentially mitigating side effects and addressing concerns about antibiotic resistance. However, its efficacy remains moderate compared to established treatments, highlighting the need for further research to optimize its formulation and therapeutic applications. Green tea represents a sustainable and biocompatible approach to periodontal therapy, supporting its integration into preventive and therapeutic oral health strategies.

## 1. Introduction

Oral health is essential for overall wellbeing as it supports critical daily functions, including eating, speaking, and social interaction. According to the WHO’s Global Oral Health Status Report (2022), approximately 50% of the world’s population—around 3.5 billion people—suffer from some form of oral disease. The most common contributors to this burden include dental caries, periodontal disease, oral cancers, and edentulism. Unhealthy diets, mainly those high in sugar, along with tobacco use and alcohol consumption, are major modifiable risk factors for these conditions [1]. Untreated dental issues can result in significant pain, tooth loss, and impaired chewing and eating, leading to nutritional deficiencies, a decline in quality of life, and reduced self-esteem. Furthermore, the costs associated with treatment place a significant economic burden on healthcare systems [2].

Periodontal disease, comprising gingivitis and periodontitis, is one of the most common oral inflammatory conditions. It affects the tissues that support and stabilize the teeth. In the initial stage, gingivitis presents with gum inflammation, bleeding, and discomfort. If left untreated, it may progress to periodontitis, destroying the periodontal ligament and alveolar bone [3]. This can lead to tooth loss, impaired mastication, and diminished self-confidence and life satisfaction. Periodontal disease is often referred to as a silent disease due to its subtle early symptoms, which can delay professional intervention. Emphasizing early diagnosis and treatment is critical to halting its progression [4].

Periodontal disease begins with the persistence of microbial biofilm on the tooth surface, eventually migrating into the surrounding periodontal pockets. This migration disrupts the balance between the host’s immune system and the microbial community within these pockets, triggering inflammation. A specific group of Gram-negative bacteria, the “Red Complex”, is strongly associated with periodontal disease. This group includes *Porphyromonas gingivalis*, *Treponema denticola*, and *Tannerella forsythia*, which are found in higher concentrations in individuals with periodontitis than those without clinical signs of the disease [5].

Advancements in molecular techniques have identified additional pathogens involved in periodontal disease. These include the Gram-positive bacterium *Filifactor alocis* and anaerobic species from the genera *Parvimonas*, *Fusobacterium*, and *Prevotella*. Among these, *Porphyromonas gingivalis* is considered the primary pathogenic bacterium in chronic periodontitis. Its colonization disrupts the host’s innate immune response and promotes inflammation, leading to shifts in the composition and quantity of the subgingival microbiota, ultimately resulting in microbial dysbiosis [6].

The tissues supporting the teeth are well-vascularized and continuously exposed to gingival crevicular fluid, which plays a vital role in maintaining equilibrium between subgingival microorganisms and the host’s immune responses. This inflammatory surveillance system ensures homeostasis in healthy individuals by recruiting neutrophils and other immune cells to prevent microbial overgrowth. However, when this balance is disrupted, it paves the way for the progression of periodontal disease [7].

Disruptions in this balance lead to the recruitment of leukocytes, primarily polymorphonuclear neutrophils (PMNs), to the site of infection, triggering inflammation. In periodontal tissues and the gingival sulcus, neutrophils are the primary inflammatory cells that accumulate in response to periodontal pathogenic bacteria within the biofilm. To combat these bacteria, neutrophils release elastase, an enzyme that damages bacterial membrane proteins. However, elastase degrades types I–IV collagen and elastin in the periodontal ligaments. This accelerates the breakdown of the extracellular matrix by activating cascades of matrix metalloproteinases (MMPs), ultimately leading to attachment loss and pocket formation. In addition to elastase, neutrophils exacerbate tissue damage by releasing MMPs and reactive oxygen species (ROS) [8]. While ROS at physiological levels can destroy periodontal pathogens and act as secondary messengers in biological processes, excessive ROS production can damage tissues and initiate a harmful cycle between the immuno-inflammatory cascade and ROS. Pathogenic bacteria and lipopolysaccharides (LPS) in subgingival plaque further aggravate the situation by activating tumor necrosis factor-alpha (TNF-α) and Toll-like receptors (TLRs) through their DNA. This triggers the release of inflammatory cytokines, which in turn promote the production of ROS by hyperresponsive PMNs [9,10].

Additionally, activated neutrophils increase the expression of receptor activators of nuclear factor kappa-B ligand (RANKL), which stimulates osteoclastogenesis and bone resorption. Activated B and T cells also contribute to this process by producing RANKL, further amplifying bone resorption in diseased gingival tissues. B cell activation results in the proliferation and maturation of plasma cells, which release cytokines such as TNF-α, transforming growth factor-beta (TGF-β), interleukin-6 (IL-6), and interleukin-10 (IL-10). TNF-α, in particular, induces MMP expression, promoting MMP-mediated destruction of periodontal tissues [8]. Depleting periodontal tissues produces oxidized proteins, inflammatory mediators, and lipid peroxides, which further activate neutrophils, fibroblasts, and macrophages, thereby increasing ROS production and perpetuating the tissue damage and inflammation cycle [9,10].

The primary approach to treating periodontal disease is maintaining proper daily oral hygiene. Regular tooth brushing and flossing are highly effective in reducing dental plaque and are the most recommended methods for maintaining oral health and minimizing the risk of developing gingivitis and periodontal disease [11,12]. Additionally, professional treatments such as supra- and subgingival scaling, root planing, air polishing (AirFlow), antibiotics, and surgical procedures may be employed when necessary [13]. To further inhibit plaque biofilm growth, various antimicrobial agents are incorporated into toothpastes and mouthwashes. Chlorhexidine (CHX) remains the gold standard for chemical plaque control due to its potent and well-documented anti-plaque properties. As a broad-spectrum antimicrobial agent, CHX effectively combats bacteria, yeasts, and viruses, making it highly beneficial for managing gingivitis and periodontal disease. However, CHX is not without its drawbacks. Prolonged use can lead to undesirable side effects such as teeth staining, dry mouth (xerostomia), altered taste sensations, and tongue numbness. Additionally, some pathogens have demonstrated the ability to develop resistance to CHX [14].

To address these limitations, there is a growing need for innovative approaches to maintaining oral health and preventing gingivitis and periodontal disease. One promising strategy involves using herbal extracts, which have been widely utilized in medicine due to their anti-inflammatory, antimicrobial, and analgesic properties. Several studies have evaluated the effectiveness of herbal mouthwashes in comparison to CHX. While some herbal formulations have shown slightly lower efficacy than CHX in controlling plaque and gingivitis, they remain valuable as prophylactic mouthwashes due to their high safety profile and minimal adverse reactions. This natural approach offers an alternative for individuals seeking gentle yet effective methods to promote oral health, highlighting the potential of herbal remedies in complementing traditional treatments for periodontal disease [15,16].

Herbal products are characterized by their complex biological activity, desirable safety profiles, cost-effectiveness, and biocompatibility. These attributes often make them a preferred choice over conventional chemical compounds, which tend to cause more adverse reactions. Plants have been used to prevent and manage various dental diseases for centuries, highlighting their historical and therapeutic significance. A systematic review by Amanpour et al. summarized multiple studies evaluating the use of herbal formulations in treating oral diseases and improving oral health. The findings revealed that herbal remedies effectively reduce dental plaque formation, manage gingivitis, and improve overall oral conditions, all while maintaining a high safety profile [17].

A multimodal approach is essential for addressing complex diseases like periodontitis. An ideal treatment formulation should offer anti-inflammatory, antimicrobial, and antioxidant properties to reduce dental plaque and limit periodontal tissue damage caused by progressive inflammation. Many plant and herbal extracts possess these beneficial properties, making them promising candidates for managing periodontitis [18]. Among the various herbal remedies, several have shown significant potential for treating periodontitis. These include *Aloe vera*, curcumin from *Curcuma longa*, *Melaleuca alternifolia* (tea tree oil), *Scutellaria baicalensis* (Baikal skullcap), *Eucalyptus globulus*, *Glycyrrhiza glabra* (licorice), *Plantago major* (plantain), and *Camellia sinensis* (green tea) [19,20]. Furthermore plants such as *Nigella sativa* [21], *Syzygium aromaticum* (clove) [22], *Calendula officinalis* [23], *Rosmarinus officinalis* [24], *Mentha species* (mint) [25], *Quercus species* (oak), and *Salvia officinalis* (sage) also present promising results in managing periodontal disease [26]. These natural extracts offer a range of therapeutic benefits and hold promise as alternative or complementary treatments in the fight against periodontitis.

## 2. The Active Compounds and Health Benefits of Green Tea

Green tea (*Camellia sinensis*), a widely cultivated plant, provides fresh tender leaves used in the production of commercial tea products. As one of the most popular beverages worldwide, tea is cherished for its delightful flavor and numerous health benefits. Based on production and fermentation methods, commercial tea products are typically categorized into three main types: non-fermented, partially fermented, and post-fermented teas. Green tea and Pu-erh raw tea serve as examples of non-fermented teas [27]. Green tea deserves special attention due to its unique properties and health benefits. Numerous studies evaluated its positive effects, such as antioxidant and anti-inflammatory [28,29], anticancer [30,31,32,33], antimicrobial [34,35,36], antidiabetic [37], neuroprotective [38], relaxing and calming [39,40], and reducing cardiovascular disease incidence [41,42]. The European Medicines Agency (EMA) also recognizes the non-fermented leaves of green tea under the traditional use regulatory pathway. Therapeutic indications include alleviating fatigue and weakness. Green tea is available in pharmaceutical forms, such as herbal or comminuted herbal substances (as herbal tea for oral use), and as herbal preparations in solid dosage forms, such as capsules containing 390 mg of powdered leaf [43].

Green tea is considered a safe product across various intakes and preparation methods. However, products containing concentrated extracts with high levels of specific components, such as epigallocatechin-3-gallate (EGCG), may require health-based guidelines to ensure safe consumption [44]. While animal toxicity studies and some case reports have suggested potential risks of liver injury from high-dose dietary supplements containing green tea catechins, food supplements with EGCG levels of up to 300 mg/day are widely considered safe [45]. Nevertheless, concerns arise when higher doses are consumed. The European Food Safety Authority (EFSA) has evaluated the effects of EGCG at doses of 800 mg/day and above as a food supplement. Data indicate that such high doses can cause a statistically significant increase in serum transaminases, which are markers of liver injury. This adverse effect has been associated explicitly with food supplements containing concentrated green tea extracts, which differ in composition, concentration, and consumption patterns from traditional green tea infusions. Notably, no hepatotoxicity was observed at doses below 800 mg/day for up to 12 months, except for a specific ethanolic extract (80% EGCG) at 375 mg/day. The 800 mg/day threshold significantly exceeds the average EGCG intake from green tea infusions in the European Union, which ranges between 90 and 300 mg/day. This distinction underscores the potential risks associated with high-dose green tea extract supplements compared to traditional green tea consumption [46]. Safety assessments, such as those conducted by Dekant et al., affirm that green tea catechins consumed through infusions and fortified beverages are safe for consumption without adverse effects [45]. Despite many health-promoting reports, a single study indicates a negative association between green tea consumption and the risk of periodontal disease among Korean adults; more studies need to be conducted to substantiate this claim [47].

### 2.1. Active Compounds of Camellia sinensis

Various factors, including cultivation methods, climate, season, and the plant’s type and age, influence green tea’s composition. The main active compounds in *Camellia sinensis* can be categorized into several groups: polyphenols, amino acids, polysaccharides, purine alkaloids, minerals, and vitamins.

#### 2.1.1. Polyphenols

Polyphenols are plant-derived secondary metabolites that help protect plants from environmental stresses such as UV radiation and pathogens. These compounds are typically categorized into flavonoids, phenolic acids, lignans, and stilbenes. Flavonoids, the most common polyphenols, are benzo-γ-pyrone derivatives and consist of phenolic and pyrane rings. They are further divided into various types: flavanols, flavones, flavonones, isoflavones, and anthocyanidins. In green tea, polyphenols primarily consist of flavonoids (mainly catechins, with fewer anthocyanins) and phenolic acids. These polyphenols are tea’s most significant bioactive components and make up about 15% to 35% of its dry weight [48].

##### Catechins

**Figure 1:**
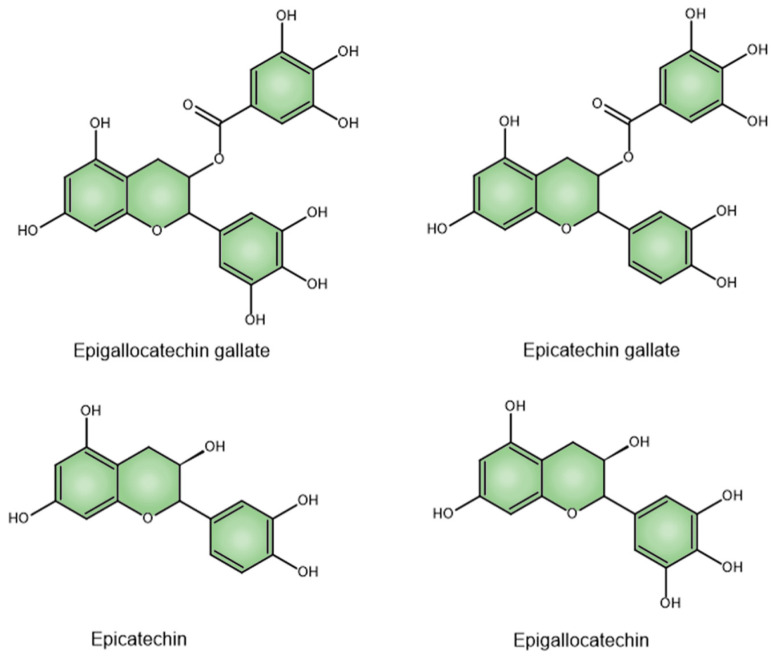
Chemical structures of EGCG, ECG, EC, and EGC.

Catechins (flavan-3-ols) make up 70% of the tea polyphenols and are the primary active constituents [48]. They make up 6–16% of the dry weight of green tea leaves. The four main catechins are (–)-epigallocatechin-3-gallate (EGCG), which accounts for about 59% of the total catechins; (–)-epigallocatechin (EGC) at approximately 19%; (–)-epicatechin-3-gallate (ECG) around 13.6%; and (–)-epicatechin (EC) at roughly 6.4% (Figure 1) [49]. Catechins are colorless, water-soluble compounds that contribute to beverages’ bitter taste and astringency [50].

##### Other Flavonoids

Green tea also contains several other types of flavonoids, including apigenin, myricetin, kaempferol, quercetin, and their glycosides [50]. Apigenin is known for its chemopreventive properties. It is recognized as an anti-proliferative, anti-inflammatory, and anti-metastatic agent [50].

##### Phenolic Acids

Fresh tea also contains various phenolic acids, which can exist independently or combine with sugar molecules to form hydrolysable tannins [48]. Examples of phenolic acids found in green tea include gallic acid, chlorogenic acid, ellagic acid, caffeic acid, p-coumaric acid, and galloylquinic acid [51,52].

Numerous studies and clinical cases have shown that tea polyphenols possess excellent antioxidant, anti-inflammatory, anti-cardiovascular disease, anti-obesity, antidiabetic, and other beneficial properties [48]. Among these polyphenols, EGCG (epigallocatechin gallate) is the predominant catechin in tea and has been the primary focus of green tea research. EGCG has attracted significant attention due to its protective effects against cancer and other diseases, including diabetes, neurological disorders, and cardiovascular diseases [53].

#### 2.1.2. Amino Acids

Tea brewing and its extract also contain amino acids. Key amino acids in tea include aspartic acid, glutamic acid, arginine, alanine, tyrosine, and theanine. Theanine, in particular, is a unique non-protein amino acid found in tea [51].

L-theanine, a distinctive free amino acid, is a crucial tea component, making up over 50% of all amino acids in the leaves of *C. sinensis* [52]. It is the most abundant amino acid, comprising about 1–2% of the dry weight of green tea leaves, and is considered the third most significant component of the dry leaf. Notably, L-theanine is the only amino acid exclusive to tea plants, contributing to the distinctive taste of green tea [54]. L-theanine is known for its numerous health benefits, including antioxidant, anti-inflammatory, neuroprotective, anticancer, metabolic regulatory, cardioprotective, hepato- and nephroprotective, immune regulatory, and anti-obesity effects [55].

#### 2.1.3. Alkaloids

Tea leaves also contain purine alkaloids, including caffeine (2–5%), theobromine, and trace amounts of theophylline [52]. Caffeine, chemically known as 1,3,7-trimethylxanthine, and theobromine, or 3,7-dimethylxanthine, stimulate the body. Caffeine acts as an antagonist to the GABA receptor, offering protective benefits against Alzheimer’s and Parkinson’s diseases. As an adenosine receptor antagonist, theobromine enhances neurotransmitter release, leading to stimulation. Both compounds block adenosine receptors, with caffeine specifically influencing cardiac activity by increasing heart rate through modulation of the adenosine A1 receptor in cardiac muscle [50].

#### 2.1.4. Saccharides

The slightly sweet taste of tea is due to the presence of small amounts of monosaccharides and disaccharides, such as glucose, fructose, galactose, and sucrose. Most carbohydrates in tea are polysaccharides like cellulose, starch, and pectin, which are not soluble in water [54].

#### 2.1.5. Volatile Compounds

Volatile organic compounds (VOCs) play a crucial role in determining tea’s aroma and overall quality. Among these, linalool and (Z)-hex-3-enal are the primary compounds responsible for the distinctive fragrance [50].

#### 2.1.6. Minerals

Tea leaves are rich in minerals, including manganese, nickel, selenium, iodine, aluminum, calcium, magnesium, sodium, and potassium, among others [50].

#### 2.1.7. Other Compounds

Besides the chemical components, green tea contains various vitamins, including vitamins B, C, and E. It also has enzymes such as glucosidases and lipoxidases. Chlorophyll and carotenoids are present as pigments [52].

### 2.2. Green Tea Compounds and Oral Health

#### 2.2.1. Antimicrobial Effect

The main active components of *C. sinensis* are catechins. The antibacterial activity of green tea catechins relies on several mechanisms, including inhibiting virulence factors like toxins and extracellular matrix, disruption of cell walls and membranes, inhibition of intracellular enzymes, induction of oxidative stress, DNA damage, and iron chelation. These mechanisms often work together, with their relative significance varying among bacterial strains. Studies on structure-activity relationships have shown that galloylated compounds, such as EGCG and ECG, exhibit the highest antibacterial activity [56].

While tea catechins have a more substantial effect on Gram-positive bacteria, they also influence Gram-negative bacteria to a lesser extent due to their protective outer membrane. Damage to the membrane impairs bacterial attachment to host cells and inhibits biofilm formation, both crucial steps in infection. Additionally, this damage prevents bacteria from secreting toxins. Catechins, especially EGCG, also inhibit enzymes involved in bacterial fatty acid synthesis, such as FabG and FabI, affecting bacterial metabolism and the production of harmful metabolites. Green tea catechins further block other essential bacterial enzymes, including protein tyrosine phosphatase and cysteine proteinases, and interfere with DNA replication by inhibiting DNA gyrase. Moreover, they can inhibit dihydrofolate reductase, preventing bacteria and yeast from synthesizing folate, and reduce bacterial ATP production by inhibiting ATP synthase [57].

Green tea catechins exhibit antibacterial properties through multiple mechanisms and enhance the efficacy of various antibiotics, making them promising agents in combating antibiotic-resistant bacterial strains. Specific antibiotics whose effects are increased by catechins include penicillins (catechins inhibit PBP2 production in MRSA, enhancing penicillin activity) [58,59]; carbapenems (galloylated catechins increase their sensitivity against methicillin-resistant *Staphylococcus aureus*; MRSA) [60,61]; monobactams (EGCG increases uptake and decreases efflux in *P. aeruginosa*) [62]; and tetracyclines (EGCG inhibits the Tet(K) efflux pump in resistant *Staphylococcus* species) [63,64].

For bacteria related to dental caries and periodontal disease, high doses of EGCG can destroy bacterial structures, while low doses inhibit virulence factors. This results in disrupted biofilms inhibited growth, reduced bacterial adhesion and aggregation, impaired nutrient absorption, and decreased tissue invasion [65]. A study by Fournier-Larente et al. found that green tea catechins could enhance the antibacterial effects of conventional antibiotics, with the most potent synergistic effect observed when combined with metronidazole. This study’s commercial green tea extract contained 98.42% polyphenols, including 47.92% EGCG, although the extraction method and DER were not defined. The authors prepared a 20 mg/mL solution of green tea extract and a 10 mg/mL EGCG solution in sterile, distilled water, which was further diluted for subsequent experiments. The green tea extract and EGCG effectively prevented *P. gingivalis* from adhering to oral epithelial cells, potentially reducing bacterial colonization in periodontal tissues. This effect may be due to catechins interacting with the bacterial membrane, altering its charge or masking adhesins crucial for attachment. Both green tea extract and EGCG demonstrated a growth-inhibitory effect against all three tested *P. gingivalis* strains. They also reduced the expression of genes related to *P. gingivalis* virulence factors, such as *fimA* (involved in biofilm formation) and *hagA* and *hagB* (linked to host colonization). The reduced expression of *fimA* aligns with previous studies showing that EGCG inhibits biofilm formation. Additionally, the green tea extract and EGCG suppressed the expression of the hem gene, which is involved in the bacterium’s ability to acquire heme from erythrocytes, a vital nutrient in periodontal pockets. Overall, it was demonstrated that green tea extract and EGCG inhibit the growth and adherence of *P. gingivalis* and reduce the expression of key virulence factors [66] (Table 1).

Khan et al. evaluated the antimicrobial activity of aqueous extracts (1 g/mL; DER 1:1) from *C. sinensis* against various clinical isolates. The extracts’ concentration ranged from 20% to 40% for Gram-positive bacteria and 35% to 60% for Gram-negative bacteria. The authors concluded that aqueous extracts of *C. sinensis* are effective against a broad spectrum of Gram-positive, Gram-negative, and fungal clinical isolates. Furthermore, the extracts demonstrated significant efficacy against drug-resistant strains, such as MRSA, *Pseudomonas aeruginosa*, and *Candida albicans* [67].

**Figure 2:**
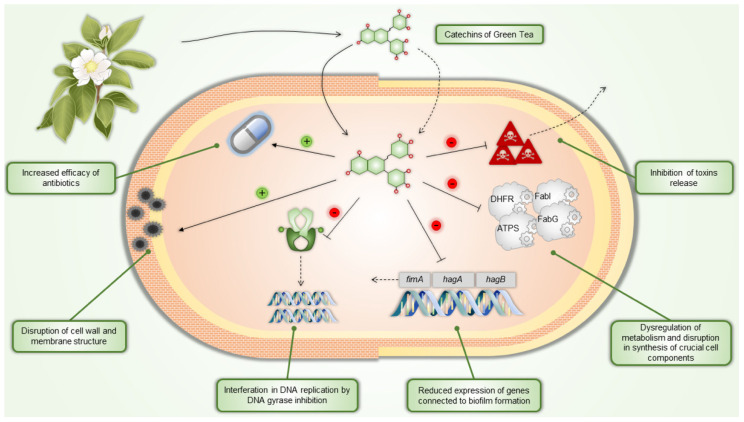
Green tea catechins’ antibacterial mechanisms of action. DHFR—dihydrofolate reductase, ATPS—ATP synthase, FabI—enoyl-ACP reductase, FabG—3-ketoacyl-ACP reductase.

Figure 2 depicts examples of the antibacterial effects of green tea catechins.

#### 2.2.2. Antioxidant and Anti-Inflammatory Effect as Protection of Bones and Tissues

In addition to their direct antimicrobial effects, tea catechins also contribute to antimicrobial activity by reducing inflammation, particularly inflammation caused by oxidative stress. They can enhance nitric oxide synthesis, inhibit the expression of C-reactive protein and other inflammatory molecules like IL-6, and suppress the production of IL-8 and hyaluronidase activity. These anti-inflammatory effects further enhance their antimicrobial potential [57].

Tea catechins have been recognized as potent antioxidants based on both in vitro and in vivo studies. Antioxidant therapy is emerging as a promising new approach for both prevention and treatment, scavenging free radicals and ROS to prevent the damage they cause [68]. ROS plays significant roles in both physiological and pathological processes, such as the initiation of periodontitis. They are involved in various signaling pathways, including MAPK, NF-kB, and PI3K/AKT, and contribute to oral diseases, inflammation, and infections [69]. Catechins effects on free radicals are diverse and include direct neutralization of ROS and reactive nitrogen species (RNS), binding to trace metals involved in the generation of free radicals (such as copper or iron), stimulation of the production of endogenous antioxidants like superoxide dismutase (SOD) and glutathione, inhibition of enzymes that facilitate ROS generation (such as glutathione S-transferase, microsomal monooxygenase, mitochondrial succinoxidase, or NADH oxidase), and also protection and regeneration of other antioxidant compounds, such as vitamins C and E. These mechanisms collectively contribute to the significant antioxidant potential of tea catechins [70]. EGCG has demonstrated significant antioxidant and free radical scavenging abilities, which various studies have supported. It has been shown to activate nuclear factor erythroid 2-related factor 2 (Nrf2), a key transcription factor that is a primary regulator of the body’s antioxidant defenses. It plays a crucial role in maintaining proper cellular redox balance by activating the expression of antioxidant enzymes that protect cells from ROS and prevent oxidative damage [71]. Furthermore, it promotes wound healing by targeting Notch, inhibiting NF-κB transcription, and reducing inflammation and ROS enzymes [72].

Catechins also hold great potential as a clinical strategy for diseases affecting bone tissue and as a therapeutic alternative for osteoarthritis and periodontitis due to their osteogenic and anti-osteoclastogenic properties, which help protect bone tissue [73]. For instance, they decrease the secretion of metalloproteinases, enzymes responsible for tissue destruction. They also improve bone density and reduce bone loss in local or systemic diseases. EGCG is believed to have a protective effect due to its antioxidant properties. It prevents or reduces inflammation and bone loss by inhibiting osteocyte apoptosis, decreasing osteoclast activity, and increasing osteoblast activity, thus balancing the RANKL/OPG ratio [74,75]. EGCG inhibits osteoclastogenesis at low concentrations through the RANK/RANKL/OPG pathway [75]. Specifically, EGCG has been shown to increase calcium levels and reduce tartrate-resistant acid phosphatase (TRAP), NF-ATc1, and IL-6 in vitro. It also lowers TNF and RANKL in human periodontal pockets and increases bone mineral density (BMD) in postmenopausal women [73].

In the study of Tominari et al., EGCG inhibited inflammatory bone resorption induced by LPS in ex vivo cultures of mouse calvarial bones. Specifically, EGCG suppressed PGE2 production in osteoblasts by downregulating the expression of COX-2, mPGES-1, and mPGES-2, and reduced RANKL expression, a key factor in osteoclast differentiation. This suppression of RANKL led to a decrease in osteoclastic bone resorption. Furthermore, LPS, a component of Gram-negative bacteria, activates TLR4 on osteoblasts, leading to inflammation and bone resorption. EGCG appears to interfere with this TLR4 signaling, reducing PGE2 biosynthesis in osteoblasts. In periodontal diseases characterized by bacterial plaque accumulation and subsequent bone destruction, EGCG has shown promise. In both in vitro and in vivo models, EGCG effectively suppressed LPS-induced alveolar bone resorption and periodontal bone loss. These findings suggest that EGCG could be beneficial in treating periodontitis [76].

Furthermore, the findings of Xu et al. offer new insights into the molecular mechanisms by which EGCG exerts its anti-osteoclastogenesis effects. The interaction between RANKL and RANK recruits TNF receptor-associated factor 6 (TRAF6), triggering downstream signaling pathways such as NF-κB, JNK, ERK, and p38. EGCG was found to inhibit the phosphorylation of IKKα/β, IκBα, and p65 and suppressed the RANKL-stimulated MAPK activation pathway. This inhibition extended to the master transcription factors NFATc1 and c-Fos, resulting in decreased levels of osteoclastogenesis-related proteins like TRAP, c-Src, and cathepsin K. Authors also claim that due to EGCG’s unique chemical structure, it likely disrupts multiple downstream entities in osteoclastogenesis. Although the detailed molecular mechanisms are still being explored, it is established that blocking the RANKL–RANK interaction is crucial for inhibiting osteoclastogenesis, and EGCG can directly bind to RANK and RANKL, disrupting this interaction. By inhibiting key signaling pathways such as NF-κB and MAPK, EGCG suppresses the expression of crucial osteoclastogenesis-related proteins, thereby hindering osteoclast differentiation and function. The authors also state that since consuming green tea has protective effects on bone health, it can potentially contribute to preventing and treating postmenopausal osteoporosis [77].

In study by Cai et al. EGCG inhibited inflammatory mediators like IL-1β, IL-6, TNF-α, and RANKL. The levels of these mediators are elevated during *P. gingivalis* infection. EGCG treatment reduced the expression of these cytokines and suppressed bone loss in mice. EGCG also regulates inflammatory pathways by suppressing NF-κB activation, which produces proinflammatory mediators. Additionally, EGCG showed antibacterial properties, disrupting the biofilm formation and membrane integrity of *P. gingivalis*. Although EGCG did not significantly reduce IL-17 levels, it still effectively reduced inflammation and alleviated bone resorption [78].

Another study conducted by Kaboosaya et al. aimed to investigate the dose- and time-dependent effects of green tea (1.5 g of green tea leaf in 60 mL of water, 3 g/60 mL, or 6 g/ 60 mL; DER 1:40, 1:20 and 1:10 respectively) on periodontitis-induced alveolar bone resorption. The results showed that EGCG, in a model of experimental ligature-induced periodontitis, prevents osteoclastic bone resorption by inhibiting RANKL expression and decreasing prostaglandin E biosynthesis in osteoblasts. Their findings indicate that green tea reduces inflammatory cells, particularly neutrophils, in the connective tissue at the junctional epithelium. This aligns with previous studies showing green tea’s inhibitory effect on osteoclastic bone resorption and osteoclastogenesis via inhibition of NF-kB, IL-6, and TNF pathways. Administration of green tea in this study showed a therapeutic effect on alveolar bone resorption in experimental periodontitis, with the most significant impact observed at a concentration of 6 g/60 mL green tea after one week [79].

Topical administration of agents like green tea extract may effectively complement mechanical treatments for periodontitis. The study of De Almeida et al. aimed to evaluate the effectiveness of commercial green tea water extract (20 mg/mL; DER and extraction method not further defined) as an adjunct to scaling and root planing (SRP) in treating periodontitis. Results indicated that the combination of SRP and green tea extract significantly reduced inflammation and alveolar bone loss compared to SRP alone. This was evidenced by lower levels of pro-inflammatory cytokines (TNF-α, IL-1ß, IL-6, IL-8), inhibition of osteoclast activity, and enhanced secretion of anti-inflammatory mediators like IL-10. Histopathological evaluations confirmed these findings, showing improved periodontal repair and reduced SRP/green tea group damage. Further, in vitro and in vivo studies supported green tea catechins’ anti-inflammatory and bone-protective effects, highlighting their potential in periodontal therapy. The study demonstrated that green tea extract as an adjunct to SRP effectively reduces inflammation and bone loss in experimental periodontitis, suggesting its potential as a valuable therapeutic option [80].

**Figure 3:**
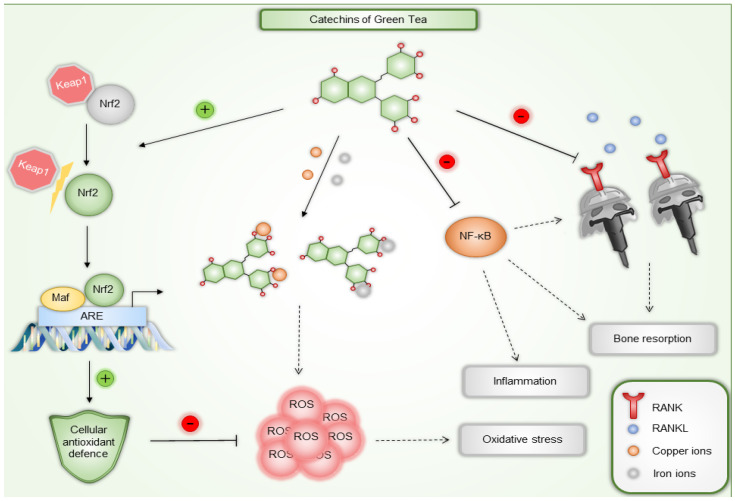
Examples of green tea catechins’ anti-inflammatory, antioxidant, and antiresorptive mechanisms of action. Besides directly reacting with ROS, green tea catechins can also activate Nrf2 transcription factor and stimulate expression of antioxidant enzymes. They can also chelate both copper and iron ions, preventing them from generating ROS. Inhibition of proinflammatory NF-κB suppresses inflammation and bone resorption. Furthermore, EGCG can disrupt RANK and RANKL interaction leading to inhibition of osteoclastogenesis and subsequently reducing bone resorption. Nrf2—nuclear factor erythroid 2-related factor 2, ROS—reactive oxygen species, ARE—antioxidant response element, Keap1—Kelch-like ECH-associated protein 1, NF-κB—nuclear factor kappa-light-chain-enhancer of activated B cells, RANK—receptor activator of nuclear factor κB, RANKL—RANK ligand.

Figure 3 and Table 1provides examples of green tea catechins’ anti-inflammatory, antioxidant, and antiresorptive effects.

**Table 1:** Basic research reports on action of green tea and its catechins.

Study	Addressed Conditions	Green Tea/ Catechins Used	Doses	Effect	Reference
Fournier-Larente et al.	*P. gingivalis* infection	Green tea extract EGCG	20 mg/mL 10 mg/mL	Inhibits biofilm formation and colonization, prevents adhesion and growth	[66]
Khan et al.	Bacterial infection (Gram-positive and Gram-negative)	Green tea aqueous extracts, (DER 1:1)	1 g/mL and further diluted	Inhibitory effect on bacterial growth	[67]
Tominari et al.	Bone resorption	EGCG	30, 60, and 90 μM (in vitro) 0.5 mg (in vivo)	Inhibition of inflammatory bone resorption induced by LPS both in vitro and in vivo	[76]
Kaboosaya et al.	Alveolar bone resorption	Green tea extract (DER 1:40, 1:20, 1:10)	1.5 g/60 mL, 3 g/60 mL, 6 g/60 mL	Prevents osteoclastic resorption, inhibits NF-kB, IL-6, and TNF pathways	[79]
De Almeida	Periodontitis treatment adjunct	Green tea extract	20 mg/mL	Combined with SRP, reduces inflammation, alveolar bone loss, IL-1ß, and TNF-α	[80]

## 3. Clinical Trials

There is an increasing number of clinical trials exploring green tea’s potential as an adjunct in preventing and treating oral diseases (Table 2). Given the global issue of antibiotic resistance and the side effects associated with agents like CHX, which are unsuitable for chronic disease management, developing new, patient- and eco-friendly agents should be encouraged [81].

Kushiyama et al. suggests a modest inverse relationship between daily green tea consumption and periodontal disease. Regularly drinking green tea during meals and breaks is a simple habit that may contribute to maintaining healthy gums. However, since the link between green tea intake and periodontal health is weak, using concentrated green tea components, like catechins, might offer more significant benefits for periodontal health [82].

The randomized controlled clinical trial results by Chopra et al. demonstrate that regular consumption of green tea is an effective and promising supplement to mechanical periodontal treatment for managing mild to moderate chronic periodontitis. The case group received green tea sachets, whereas the control group received placebo sachets containing cellulose. Participants were instructed to steep the sachets in 240 mL of hot water for 1 to 2 min and consume two cups daily over 12 weeks. When combined with mechanical periodontal treatment, regular intake of green tea significantly boosted antioxidant levels in gingival crevicular fluid (GCF) and plasma. This increase in local antioxidant capacity was more noticeable in GCF, likely due to the catechins in green tea binding to oral mucosal receptors, salivary proteins, and acquired pellicle. Since GCF is the first fluid interacting with periodontal tissues, maintaining a high local antioxidant level is crucial for effective treatment. The antioxidant capacity of GCF and plasma was significantly higher in the green tea group, eight times and six times greater, respectively, than in the control group. Flavonoids of green tea promoted superior and rapid healing of gingival and periodontal tissues through potent anti-inflammatory, astringent, and anti-plaque effects [83].

The study by Rezvani et al. has shown that green tea consumption (2 cups of green tea, each containing 25 g of green tea leaves, for 6 weeks) can improve clinical indices in chronic periodontitis patients by reducing periodontal pocket depth (PPD) and bleeding. Specifically, daily consumption of green tea for six weeks significantly reduced salivary IL-1β levels. Additionally, green tea catechins inhibited IL-1β production and other inflammatory processes. The study indicates systemic green tea consumption can enhance periodontal health by lowering IL-1β levels, suggesting a beneficial dietary change for periodontitis management [84].

Hrishi et al.’s study evaluated the impact of a green tea dentifrice on gingival inflammation and periodontal disease severity when used alongside SRP in treating chronic periodontitis, compared to a fluoride-triclosan dentifrice. The toothpaste used contained 1.4% (*w*/*w*) commercial green tea extract (60–90% EGCG; DER and extraction method not further defined). Thirty patients with mild to moderate chronic periodontitis were randomly assigned to either a test group (green tea dentifrice) or a control group (fluoride-triclosan dentifrice) after initial SRP. Clinical parameters like gingival index (GI), plaque index (PI), bleeding on probing (BOP), probing depth (PD), and clinical attachment level (CAL) and biochemical parameters including total antioxidant capacity (TAOC) and glutathione-S-transferase (GST) activity were recorded at baseline and four weeks post-SRP. The study found that green tea dentifrice significantly improved GI, BOP, CAL, TAOC, and GST levels compared to the control dentifrice, with no adverse effects except mild teeth staining in one subject. These findings suggest green tea dentifrice as a beneficial adjunct to SRP in enhancing periodontal therapy outcomes [85].

A clinical trial conducted by Rattanasuwan et al. evaluated the effect of a thermosensitive green tea gel as an adjunctive treatment in patients with chronic periodontitis. A green tea gel containing *C. sinensis* extract was developed using the extract containing no less than 80% total catechins. The gel was a thermosensitive hydroalcoholic formulation with a brown color and consisted of 12% *w*/*w* green tea extract (DER and extraction method no further defined). The gel, applied after scaling and root planing, significantly reduced probing pocket depth (PPD) and bleeding on probing (BOP) compared to baseline, but no additional benefit in PPD reduction was observed between the green tea gel and placebo groups. Both groups showed improved attachment levels, but no significant difference in CAL was found. The green tea gel reduced gingival inflammation more effectively than the placebo, likely due to its antioxidant and antibacterial properties. Green tea gel demonstrated a comparable reduction in BOP to other antimicrobial treatments like tetracycline. However, it had no significant impact on plaque reduction. In conclusion, green tea gel appeared to be a useful adjunct to non-surgical periodontal treatment, particularly in reducing gingival inflammation and bleeding [86].

A systematic review by Gartenmann assessed the clinical effectiveness of topically applied green tea catechins as a natural addition to SRP, comparing it to SRP alone or with a placebo. The authors evaluated the efficacy of 1% green tea gels, 12% green tea gel, and tea catechin strips. The primary outcome measured was the reduction in PPD. Five randomized controlled trials with a split-mouth design included green tea catechins as an adjunct to SRP. The results showed that the adjunctive use of green tea catechins led to an average PPD reduction of 0.74 mm, favoring the test group. While systemic antibiotics have proven successful in treating periodontal disease, their use is associated with antibiotic resistance and systemic side effects. Therefore, locally applied natural alternatives like green tea catechins are of increasing interest due to their local anti-inflammatory effects. The clinical benefits observed align with previous reviews on other antimicrobial agents used alongside SRP. The use of green tea catechins as an adjunct to SRP was found to provide a beneficial reduction in PPD compared to SRP alone or with a placebo. However, because of the considerable variability in the data and potential risks of bias, the authors claim that these findings should be interpreted carefully [87].

Another systematic review and meta-analysis of Mazur et al. thoroughly examined the results of randomized control trials on the use of green tea and its effects on gingivitis, periodontitis, and tooth decay. It found that green tea positively influences gingival inflammation and periodontitis, providing sufficient evidence to support its use in preventing and treating periodontal disease. However, the evidence is currently insufficient to recommend green tea for managing dental caries. Although the results are promising, the authors claim that green tea cannot fully replace CHX, which remains the recommended treatment for gingivitis and periodontitis [81].

Similarly, a systematic review of Tafazoli et al. shows that most studies highlight the benefits of green tea. Therefore, they state that tea mouthwashes can be recommended for oral disinfection, dental plaque removal, and oral pain relief, supported by higher levels of evidence. A notable advantage of these formulations is their impressive safety profile, making them superior to synthetic chemical mouthwashes [88].

In the study of Rassameemasmaung et al., subjects with gingivitis used it twice daily for four weeks to evaluate the green tea mouthwash’s effect on oral malodor caused by volatile sulfur compounds (VSC) levels and gingival inflammation. Green tea mouthwash was a hydroalcoholic solution containing green tea extract (>80% total catechins). The study found a 38.61% reduction in VSC levels, significantly different from baseline and the placebo, likely due to the antimicrobial activity of green tea catechins against *P. gingivalis* and their ability to neutralize VSC [89].

**Table 2:** Clinical trials utilizing green tea.

Study	Year	Disease	Subjects	Intervention	Duration	Outcome	Reference
Rezvani et al.	2022	Chronic periodontitis	30	2 cups a day (each cup contained 25 g of green tea leaves) following SRP treatment	6 weeks	Reduction of IL-1β levels	[84]
Chopra et al.	2016	Mild to moderate chronic periodontitis	120	2 cups daily of green tea beverage over 12 weeks as an adjunct to SRP	12 weeks	Increase in antioxidant levels in GCF and plasma; improvements in GI, PI, BOP, and CAL	[83]
Hrishi et al.	2016	Mild to moderate periodontitis	30	Green tea toothpaste containing 1.4% green tea extract (60–90% EGCG content) as an adjunct to SRP	4 weeks	Improvements in GI, BOP, CAL, TAOC and GST levels on intra- and intergroup comparisons at 4 weeks	[85]
Rattanasuwan et al.	2014	Chronic periodontitis	48	Green tea thermosensitive hydroalcoholic gel containing 12% of green tea extract (no less than 80% total catechins) as an adjunct to non-surgical periodontal treatment	6 months	Reduction of PPD and BOP, improvement in attachment levels reduced gingival inflammation	[86]
Rassameemasmaung et al.	2013	Gingivitis, oral malodor	60	Hydroalcoholic green tea mouthwash	4 weeks	Reduction of VSC level in gingivitis subjects without causing remarkable side effects	[89]

PPD—probing pocket depth, BOP—bleeding on probing, GI—gingival index, CAL—clinical attachment level, TAOC—total antioxidant capacity, GST—glutathione-S-transferase activity, VSC—volatile sulfur compounds, SRP—scaling and root planning, GCF—gingival crevicular fluid.

## 4. Future Perspective

Green tea has demonstrated multifaceted benefits in oral health due to its antioxidant, anti-inflammatory, and antimicrobial properties. However, the full potential of green tea in dentistry is far from being realized. Future research and application may expand its use in the following directions:

1. Development of Specialized Green Tea-Based Products—Integrating green tea compounds into dental care products, such as toothpaste, mouthwash, gels, and chewing gums, holds significant promise. Future innovations may focus on increasing the bioavailability of active components like epigallocatechin gallate (EGCG) to ensure sustained effects. Encapsulation technologies or controlled-release formulations could be used to enhance the effectiveness of these products.
2. Green Tea as a Natural Alternative to Conventional Treatments—Green tea can replace or complement existing chemical agents like chlorhexidine, reducing side effects such as staining and taste alteration. Long-term studies must establish its efficacy as a primary treatment modality in managing conditions like gingivitis and periodontitis.
3. Synergy with Probiotics and Prebiotics—Emerging research suggests that green tea catechins can positively interact with beneficial oral microbiota. Exploring the synergy between green tea and probiotics could lead to innovative solutions for maintaining oral microbiome balance and enhancing resistance to pathogenic bacteria.
4. Application in Dental Implants and Biomaterials—Green tea’s antimicrobial and osteoprotective properties make it a potential candidate for coating dental implants or other biomaterials. Such applications may reduce the risk of peri-implantitis and promote bone integration, extending the longevity of dental implants.
5. Role in Personalized Oral Health Care—With genomics and personalized medicine advancements, green tea extracts could be tailored to individual patient needs. Variations in oral microbiota and genetic predispositions could guide the formulation of green tea-based therapeutics for targeted interventions.
6. Green Tea in Community Oral Health Programs—Given its affordability and widespread availability, green tea could be integrated into public health initiatives, particularly in low-resource settings. Programs encouraging the use of green tea infusions for oral rinsing may reduce the burden of oral diseases in underserved populations.
7. Exploration of Nano-Formulations—Nanotechnology offers an exciting avenue for enhancing the delivery and effectiveness of green tea compounds. Nanoemulsions, liposomes, or nanoparticles carrying EGCG could improve penetration into periodontal pockets or other hard-to-reach areas.
8. Long-Term Safety and Regulatory Approvals—While green tea is generally considered safe, concentrated extracts require further safety evaluations for widespread clinical use. Future research must focus on establishing clear dosage guidelines and obtaining regulatory approvals to ensure safe and practical application.

## 5. Conclusions

Green tea (Camellia sinensis) demonstrates substantial potential as a natural, sustainable, and innovative approach to managing periodontal disease. Its bioactive compounds, particularly epigallocatechin-3-gallate (EGCG), exhibit potent antioxidant, anti-inflammatory, and antimicrobial properties, improving oral health outcomes. Studies have shown that green tea consumption and topical applications can reduce periodontal pocket depth (PPD), inflammation, and bleeding, supporting its role as an adjunct to conventional treatments such as scaling and root planing (SRP).

Clinical trials and systematic reviews suggest that green tea-based products—including dentifrices, mouthwashes, and gels—can enhance periodontal therapy by modulating inflammatory pathways, inhibiting bacterial adhesion, and reducing oxidative stress. These benefits position green tea as a viable alternative to synthetic antimicrobial agents like chlorhexidine, potentially minimizing side effects such as tooth staining and altered taste perception. However, while green tea shows promise, it has not yet demonstrated the same level of efficacy as established chemical treatments.

Future research should optimize green tea formulations, explore advanced delivery systems such as nanoencapsulation, and evaluate its long-term safety for widespread clinical use. Investigating its synergistic effects with probiotics and biomaterials may expand its applications in periodontal therapy and implantology. As interest in natural alternatives grows, green tea stands as a compelling candidate for integration into preventive and therapeutic oral health strategies, with the potential to benefit both individual patients and broader public health initiatives.

## Data Availability

Data are contained within the presented article.
